# Is *Candida auris* sexual?

**DOI:** 10.1371/journal.ppat.1009094

**Published:** 2020-12-17

**Authors:** Zoe K. Ross, Alexander Lorenz

**Affiliations:** 1 Institute of Medical Sciences (IMS), University of Aberdeen, Aberdeen, United Kingdom; 2 MRC Centre for Medical Mycology, Department of Biosciences, University of Exeter, Exeter, United Kingdom; Vallabhbhai Patel Chest Institute, INDIA

Sexual reproduction and related processes play a somewhat limited but important role in generating genetic diversity in *Candida* species and other fungal pathogens. These processes are also thought to be an important contributor to the evolution of pathogenicity and drug resistance. *Candida auris* is a recently emerged, human-pathogenic yeast causing nosocomial outbreaks all over the globe [1]. It can cause serious blood stream infections with the complication that isolates are typically resistant to the available antifungal therapies; mortality rates are approximately 60% [2]. Genetic diversity is likely a major driver of its pathogenesis and virulence features. Here, we discuss which mechanisms could be behind the genetic diversity observed between *C*. *auris* isolates. Specifically, our review examines the evidence around sexual reproduction in this fungus.

## How do fungal pathogens create genetic diversity?

Fungal pathogens are able to create genetic diversity in multiple ways. Some have true meiotic cycles that generate diversity via homologous recombination, while others have evolved mechanisms of producing diverse offspring that do not depend on meiosis.

*Candida albicans* has a parasexual cycle, where fusion (mating) of 2 diploid cells is followed by concerted chromosome loss, rather than meiosis, to result in viable, but often aneuploid, progeny. Parasex generates genetic diversity and enables adaptation to stressful environments [3–5]. Although meiosis has not been observed in *C*. *albicans*, a complete meiotic cycle has been identified in the distantly related *Candida* (*Clavispora*) *lusitaniae*, a haploid yeast that can form spores through mating and meiosis [6]. *C*. *lusitaniae* often produces aneuploid progeny during meiosis, which most likely confer a selective advantage [6]. The pathogenic basidiomycete *Cryptococcus neoformans* is also capable of generating genetic diversity via chromosome copy number variations and ploidy changes, as unisexual meiosis (see below) often results in aneuploid and diploid spores [7]. Chromosome copy number variation (aneuploidies) are a means of creating diversity, as has been found in many fungal species [3,6,8]. Aneuploidies can arise by parasexual, asexual, and sexual mechanisms [7,9]. Importantly, aneuploidies can confer resistance to antifungal drugs by altering gene dosage, e.g., copy number variations (of the left arm) of chromosome 5 in *C*. *albicans* confer resistance to fluconazole [10]. The higher dosage of 2 genes on chromosome 5, *ERG11* and *TAC1*, contributes to an increase in production of the azole drug target Erg11, and higher drug efflux activity via increased expression of Tac1-regulated efflux pumps; notably some, but not all, copies of *TAC1* also were mutant expressing a hyperactive allele [10].

**Fig 1:**
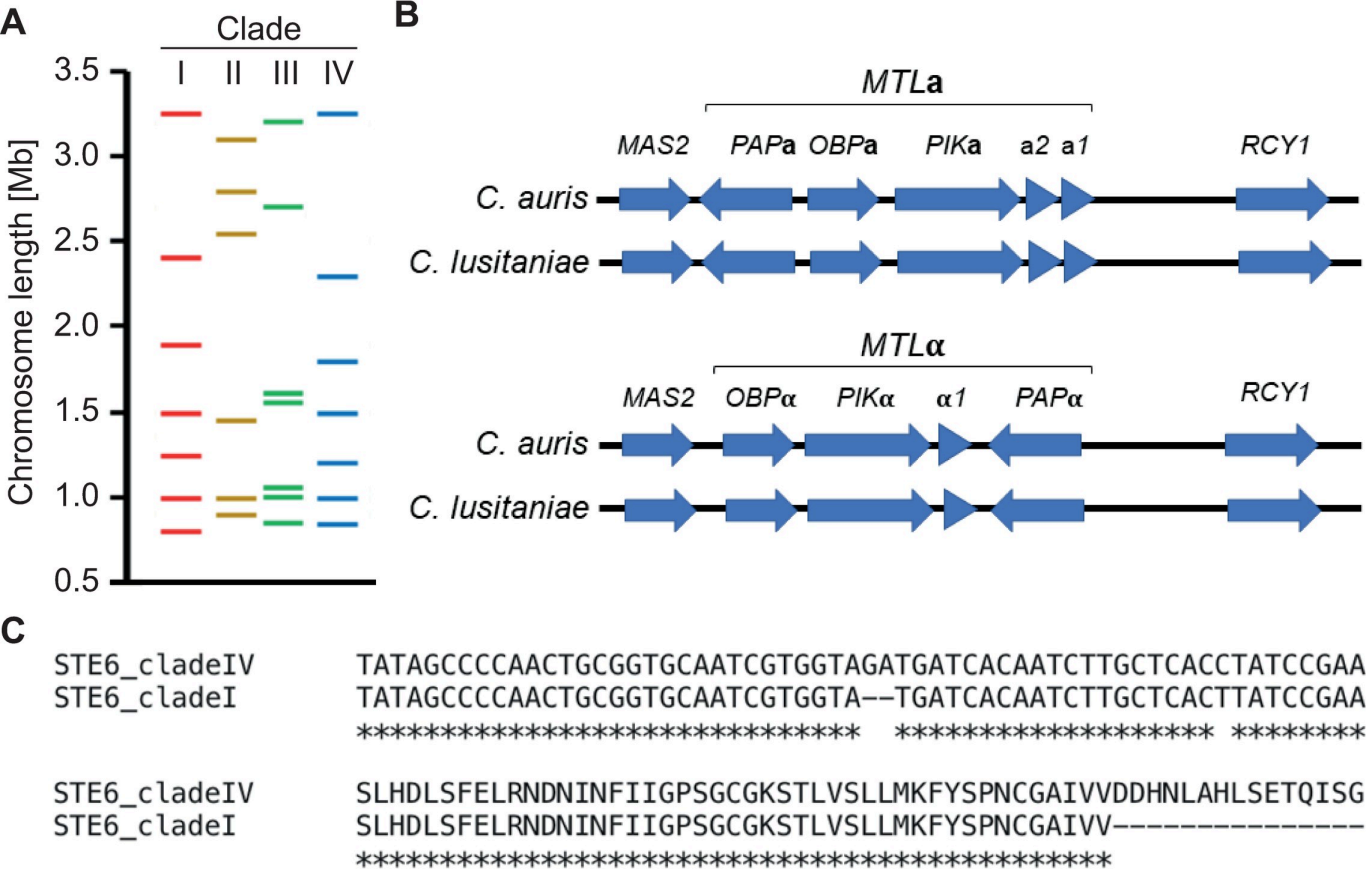
Chromosomal and genetic features of *C*. *auris* related to sexual reproduction. (A) Length and number of chromosomes of 1 isolate from each of the 4 main *C*. *auris* clades as measured by pulsed-field gel electrophoresis (strains representing clades are: clade I, UACa1/470026; clade II, UACa18/B11220; clade III, UACa20/B11221; clade IV, UACa22/B11244) [14]. (B) The mating type locus regions *MTL***a** and *MTL*α are conserved between *C*. *auris* and *C*. *lusitaniae* [26]. **(**C) ClustalΩ (https://www.ebi.ac.uk/Tools/msa/clustalo/) [38] alignments of the *STE6* nucleotide sequences (top) from a clade I and a clade IV isolate, showing the 2-nucleotide deletion in clade I; and of the translated sequences (bottom) showing the premature stop codon in the clade I isolate at position 421 generated by the 2-nt deletion [36].

Karyotype variability, including chromosome rearrangements, is common in fungi and could be a basis for genetic diversity leading to phenotypes with enhanced fitness. A wide range of species, including *Malassezia* spp., *Fusarium* spp., and *Candida glabrata*, have highly variable karyotypes that are apparently well tolerated [11–13]. Genetically identical *C*. *auris* isolates from a hospital outbreak had very similar karyotypes (except for chromosomes bearing the rRNA gene arrays which showed some size differences), suggesting that genome rearrangements do not play a major role in quickly establishing genetic variability within individual outbreaks [14]. However, passaging *C*. *auris* through several rounds of various stresses generated massive karyotype changes [14]. Moreover, the variation in karyotype between *C*. *auris* isolates from different clades would indicate that genome rearrangements are indeed a potential mechanism to generate variation (Fig 1A), as has been described for other *Candida* species. For example, studies in *C*. *albicans* have shown that chromosome rearrangements occurring after 1 passage through a mouse model are able to generate genetic and phenotypic diversity [15]. Similarly, chromosome rearrangements have also been identified in *C*. *glabrata* from sequential blood stream isolates [16].

The *C*. *auris* clades differ from each other genetically by thousands of single nucleotide polymorphisms (SNPs), yet within each clade, independent clonal expansions typically take place within an outbreak [2]. This population structure, characterised by distinct and highly variable clades that are distributed worldwide and clonal expansions of a single genotype within individual outbreaks, is puzzling and suggests that the clades emerged independently. The *C*. *auris* clades also differ in genome organisation as structural rearrangements have been identified between the clades [17]. The origins of the variability seen within *C*. *auris* are not yet known. Importantly, determining the origins of genetic diversity in this dangerous human pathogen will potentially elucidate evolutionary mechanisms behind its virulence and antifungal drug resistance. This raises the question whether this is potentially related to (para)sexual outcrossing of strains to generate new genotypes.

## Does *C*. *auris* have a complete mating type locus?

Mating types are the sex-determining genetic loci of fungi. Most fungi, including the *Candida* clade, have a mating type locus (*MAT*) or mating type-like locus (*MTL*) that occurs in 2 idiomorphs, *MAT***a** and *MAT*α (or *MTL***a** and *MTL*α). Generally, mating is only possible between cells of opposite mating types. However, there are exceptions to this rule.

The *MTL* loci in most diploid *C*. *albicans* isolates are heterozygous (***a***/*α*); therefore, these isolates were thought to never sexually reproduce as phenotypic switching from a ‘white’ to the mating-competent ‘opaque’ form is blocked in these isolates. Usually, only isolates that are homozygous at the *MTL* locus are able to switch into the opaque form [18], although *MTL*-heterozygous isolates can switch under certain conditions [19]. It was later discovered that the rare isolates that were homozygous at the *MTL* locus could form cell fusion products with isolates homozygous for the opposite mating type [18,20]. After mating, the resulting tetraploid *C*. *albicans* fusion products undergo concerted chromosome loss instead of meiosis to generate progeny often harbouring complex aneuploidies [5]. Parasex in *C*. *albicans* has the capability to produce progeny that have enhanced virulence and, in some cases, increased resistance to fluconazole, making this a clinically relevant process [4].

*Cryptococcus neoformans* was described as capable of forming basidia (the generative cell type of Basidiomycetes) more than 40 years ago [21]. However, almost all *Cryptococcus neoformans* isolates are *MAT*α (>99%); its mating type locus covers >100 kb of sequence, making it one of the largest in the fungal kingdom. Importantly, unisexual reproduction (a.k.a. haploid fruiting) between 2 *MAT*α isolates apparently plays a major role, indicating that an unequal distribution of mating type idiomorphs in a population or species does not preclude sexuality [7,22]. It is speculated that unisexual reproduction in *Cryptococcus neoformans* benefits the species as it prevents deleterious mutations from accumulating and can also yield progeny with enhanced fitness [8,23]. Unisexual mating can also occur in *C*. *albicans* and *Candida tropicalis*, but only in the presence of the opposite mating pheromone [24,25].

Investigation into the *MTL* loci of *C*. *lusitaniae* and *C*. *auris* revealed a highly conserved gene order, orientation, and synteny between these 2 closely related species (Fig 1B) [26,27]. Genome annotations have identified both mating types in *C*. *auris* and they appear to be clade-specific. So far, all sequenced clade I and clade IV isolates are *MTL***a** and all clade II and III isolates are *MTL*α [26]. Isolates of opposite mating types are yet to be found within the same clade. However, occasionally *C*. *auris* strains with opposite mating types are found in the same location, namely Canada, Kenya, the United Kingdom, and the United States of America [28,29]. The latter finding suggests that there could be a clinically relevant danger of sexual interaction producing a super-resistant or super-virulent strain.

## What is the evidence for sexuality in *C*. *auris*?

Genome sequencing data revealed that *C*. *albicans* has orthologs for most of the genes involved in mating and sporulation in *Saccharomyces cerevisiae* [30]. This raised the question whether *C*. *albicans* may be sexual and resulted in the discovery of parasex (see above). *C*. *lusitaniae* is able to carry out meiosis despite missing a full ‘meiosis toolkit’ [6,31]. Key meiotic genes are conserved between the species of the *Candida haemulonii* complex (*C*. *haemulonii*, *Candida duobushaemulonii*, *Candida pseudohaemulonii*, *C*. *auris*) and *C*. *lusitaniae* [26]. Thus, *C*. *auris* and its closest relatives should have sufficient mating and meiosis factors to support a sexual cycle. Indeed, a complete mating locus and both mating types exist in *C*. *auris*, strengthening the evidence that *C*. *auris* may be capable of mating and meiosis, or at least mating and concerted chromosome loss (parasex). So far, mating could not be observed in *C*. *haemulonii* and *C*. *duobushaemulonii* [32]. Intriguingly, an investigation into transporter family proteins in *C*. *auris* identified a mutation in *STE6* in clade I isolates. Ste6 (Hst6 in *C*. *albicans*) is an ABC family transporter which is only expressed in *MAT***a** (*MTL***a**) strains and exports the **a**-factor pheromone in *S*. *cerevisiae* and *C*. *albicans* [33–35]. In *S*. *cerevisiae*, the **a**-factor and its export via Ste6 is essential for mating [33]. The *STE6* homolog in *C*. *auris MTL***a** clade I isolates is missing 2 nucleotides at positions 3,309 and 3,310, while in *MTL***a** clade IV isolates and *MTL*⍺ strains, this open reading frame is complete [36]. The 2 missing nucleotides result in a premature stop codon at AA421 of AA1,225 and therefore, a truncated and likely nonfunctional protein (Fig 1C). This would render clade I *MTL***a** strains sterile due to an inability to export **a**-factor. We cannot exclude the possibility that *C*. *auris*, similar to *C*. *albicans* and *C*. *tropicalis* [24,25], could undergo unisexual reproduction with isolates from within the same clade.

Furthermore, for meiosis to produce viable progeny, pairing and recombination of homologous chromosomes is required [37]. Therefore, any karyotypical changes (chromosome rearrangements) between isolates will likely have a negative impact on the viability of any progeny. The karyotype differences between *C*. *auris* clades (Fig 1A) [14,26] make it unlikely that the extant clinical strains of *C*. *auris* successfully intermingle. However, these differences might not restrict parasexual mechanisms. To determine whether *C*. *auris* is sexual, it is of the utmost importance to identify its environmental reservoirs, where different *MTL* idiomorphs within a population might exist. It would appear that, based on current data, there is no threat to healthcare of *C*. *auris* mating and creating diversity in a clinical context.
